# Accounting for Material Changes in Decellularized Tissue with Underutilized Methodologies

**DOI:** 10.1155/2021/6696295

**Published:** 2021-05-31

**Authors:** Ryan A. Behmer Hansen, Xinming Wang, Gitanjali Kaw, Valinteshley Pierre, Samuel E. Senyo

**Affiliations:** Department of Biomedical Engineering, Case Western Reserve University, Cleveland, OH, USA

## Abstract

Tissue decellularization has rapidly developed to be a practical approach in tissue engineering research; biological tissue is cleared of cells resulting in a protein-rich husk as a natural scaffold for growing transplanted cells as a donor organ therapy. Minimally processed, acellular extracellular matrix reproduces natural interactions with cells in vitro and for tissue engineering applications in animal models. There are many decellularization techniques that achieve preservation of molecular profile (proteins and sugars), microstructure features such as organization of ECM layers (interstitial matrix and basement membrane) and organ level macrofeatures (vasculature and tissue compartments). While structural and molecular cues receive attention, mechanical and material properties of decellularized tissues are not often discussed. The effects of decellularization on an organ depend on the tissue properties, clearing mechanism, chemical interactions, solubility, temperature, and treatment duration. Physical characterization by a few labs including work from the authors provides evidence that decellularization protocols should be tailored to specific research questions. Physical characterization beyond histology and immunohistochemistry of the decellularized matrix (dECM) extends evaluation of retained functional features of the original tissue. We direct our attention to current technologies that can be employed for structure function analysis of dECM using underutilized tools such as atomic force microscopy (AFM), cryogenic electron microscopy (cryo-EM), dynamic mechanical analysis (DMA), Fourier-transform infrared spectroscopy (FTIR), mass spectrometry, and rheometry. Structural imaging and mechanical functional testing combined with high-throughput molecular analyses opens a new approach for a deeper appreciation of how cellular behavior is influenced by the isolated microenvironment (specifically dECM). Additionally, the impact of these features with different decellularization techniques and generation of synthetic material scaffolds with desired attributes are informed. Ultimately, this mechanical profiling provides a new dimension to our understanding of decellularized matrix and its role in new applications.

## 1. Extracellular Matrix and Its Importance in Tissue Engineering

### 1.1. Conceptualizing the Extracellular Matrix

Extracellular matrix (ECM) is a complex structure which physically supports cells in virtually every tissue in the body. ECM is generally organized into two distinct strata: the basement membrane which anchors adherent cells to the extracellular environment, and the interstitial matrix, which acts as a scaffold for the extracellular environment. Extracellular glycoproteins (such as type IV collagen, fibronectin, and laminin) comprising the basement membrane are organized in specialized sheets that interface directly with adhesive cells via sites for binding of cellular transmembrane integrins. The basement membrane superimposes on the thicker interstitial matrix. The interstitial matrix includes proteoglycans that embed fibrous structural proteins (collagens and elastins) and polysaccharides that provide structural constraints to tissues. The layered fibrous components described above provide an integral structure for cells in tissue.

The ECM is also a globular system which physically embeds soluble molecular factors (e.g., TGF-*β*), acts as a molecular sieve for diffusive factors, and transmits mechanical cues which mediate diverse biological action. It thereby affects processes such as cellular adhesion, cellular differentiation, inflammation, tissue remodeling, and intercellular signaling. Accordingly, the sieve properties of ECM are currently investigated as a druggable target for enhancing anticancer medication efficacy against solid tumors by increasing porosity, a strategy termed “stress alleviation” [1]. ECM signaling facilitates cellular homeostasis and plays a role in adaptive processes. The characterizations of these ECM-derived influences, as well as their effect on cell behavior, are a critical area of research for ECM-based bioengineering applications such as targeted drug delivery [2] and artificial platelet therapy [3].

The ECM is the primary “microenvironment” of a cell collectively with soluble factors, insoluble proteins, and neighboring cells. As evidence for the importance of this, cells cultured *in vitro* without ECM ligands exhibit suppressed metabolic function, loss of morphology, and decreased viability [4–8]. Reintroduction of cues from the microenvironment is perceived to maintain or reactivate natural cellular activity.

### 1.2. Understanding Cell-Microenvironment (Cell-ECM) Interactions

The ECM is a dynamic material that changes significantly (i.e., “remodels”) with tissue development [9], disease [10, 11], and even exercise [12]. ECM remodeling involves both biochemical and mechanical alterations maintained in biophysical feedback loops. These feedback loops are sustained by biochemical and mechanical signaling between cell and cell-microenvironment. Research in biomechanics and biophysics have broadened our understanding of ECM mechanobiological changes and inform the impact on subsequent cellular activity. Material properties of the ECM such as structure, topography, and viscoelasticity instruct cell behavior through mechanical interaction, and the nature of this mechanical interaction is modulated by cellular protein deposition, cell contraction, and cell-mediated ECM degradation. Examples of cell regulation by substrate mechanics may be found in a variety of contexts, including during tissue development, repair, tumor initiation, and disease progression as summarized in a comprehensive review by Janmey et al. [13].

**Figure 1:**
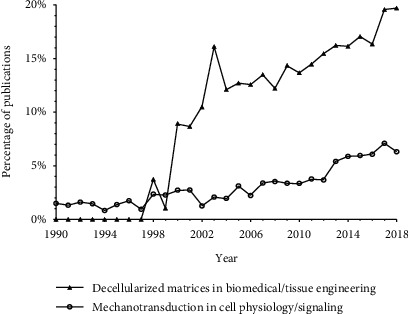
Percentage of biomedical/tissue engineering studies that incorporate decellularized matrix, by year: determined using PubMed search algorithm, with terms “decellularization”, “decellularized”, “acellular matrix”, “biological scaffold”, “acellular scaffold”, “matrix biomaterial”, “ECM”, “dECM”, “decellularized matrix”, “extracellular matrix”, “decellularized tissue”, “tissue engineering”, or “biomedical engineering”. The popularity of decellularized matrix within the field of tissue engineering has been steadily increasing since the beginning of the century. Also shown is the percentage of cell physiology/signaling papers that include mechanotransduction, by year: determined using PubMed search algorithm, with terms “mechanotransduction”, “mechanotransduction pathways”, “mechanotransduction mechanism”, “mechanosignaling”, “mechanotransductive signaling”, “mechanoreceptors”, “cellular mechanotransduction”, “cell force transduction”, “cell force transfer”, “cell force transmission”, “cell force response”, “cell mechanical”, “cell physical”, “cell communication”, “cell biology”, or “cell and tissue based therapy/physiology”. Research studies with “decellularized matrix”-related terms have increased substantially since the research area began to gain traction in the late 20th century. All searches were completed on February 22, 2020.

The importance of structural and mechanical cues provided by the ECM has direct implications for the usefulness of isolated biological scaffolds and has been comprehensively reviewed in recent times. For the interested reader, we suggest “Mechanotransduction and extracellular matrix homeostasis” [14], “Integrin-mediated mechanotransduction” [15], and “Review of cellular mechanotransduction” [16]. Also relevant for cell-substrate interactions is the review on ECM physical cues, “Extracellular matrix elasticity and topography: material-based cues that affect cell function via conserved mechanisms” by Janson and Putnam [17]. The mechanisms of mechanotransduction continue to draw attention in recent years due to their importance (see Figure 1). The inception of decellularized matrix as a tangible approach to studying cell-matrix interactions and as a candidate scaffold for tissue engineering has proven promising (as will be discussed further in Section 3). There are several techniques commonly applied to the characterization of ECM including, but not limited to, western blot methods and immunohistochemistry. These two common techniques, however, are not the most suitable for characterizing critical structural and mechanical ECM cues.

### 1.3. A Great Opportunity: Extracellular Matrix Characterization by Mechanical Testing

There are several mechanical measurement techniques which have been established and validated in nonbiologically oriented fields such as polymer science and material engineering. Creative adaptation of these techniques to evaluate cell-matrix interactions and tissue scaffolds is not often done, but has proven informative in a few pioneering studies. Key considerations related to tissue mechanics, decellularized matrix, and the evaluation of these entities through novel applications of analytic techniques such as FTIR, AFM, and DMA are highlighted in this review.

## 2. ECM Mechanics at Different Scales

In general, different analytic techniques will offer different resolutions. Thus, the scale of interest plays an important role in determining a most suitable analytic technique. Recognizing this, we conceptualize ECM physical properties in two distinct, yet related contexts: (1) bulk material characteristics and (2) local physical properties at the scale of the cell. In this section, these two contexts will be described. The descriptions provided here will motivate discussion of different analytic techniques and their applicability later in this review.

### 2.1. Bulk Tissue Characteristics

Compliance (the inverse of stiffness) is an important parameter in biologic contexts. Bulk tissue compliance from a clinical perspective is a significant factor for therapeutic success as it impacts both cell biology and overall functionality of a tissue. Prosthetic implants (e.g., teeth and skeleton) are designed to match the mechanical loads of their target tissue for stability and tissue integration. Contact lenses similarly rely on elasticity and water permeability for functionality on the eye. Conversely, the mechanical state of tissue is recognized to influence disease progression. For example, patients with emphysema suffer from difficulty with exhalation because of high lung compliance and poor elastic recoil [18]. The high compliance of emphysematous tissue also results in alveolar collapse, encumbering inhalation [19]. In the case of lung fibrosis, ventilation and gas exchange are impaired because of decreased lung compliance and poor expansion [20, 21]. These previous two examples demonstrate that there exists some optimal range for pulmonary tissue compliance. In the heart, venous and ventricular compliance are both key parameters in the determination of cardiac function [22, 23]. Stiffer aortas are associated with heart failure-associated rehospitalization in patients with dilated cardiomyopathy [24]. *In vivo* introduction of bioengineered tissues with inappropriate bulk mechanical properties will unfortunately be accompanied by introduction of clinical symptoms.

Tissue engineering approaches are numerous and diverse. The establishment of strategies to restore function to damaged tissues is an important goal of many efforts, and ensuring suitable bulk mechanical features is a crucial aspect of the design process. This is evident in the previous examples listed above. When done correctly, implementing designs with appropriate physical and mechanical aspects results in better biomedical outcomes. For example, graft failure is a visible complication of skin grafts that may be decreased by the matching of graft to tissue properties in order to reduce shear or fracture [25]. Similarly, lost myocardial mechanical stability and cardiac output may be restored postinfarct with proper physical material integration. Emerging postmyocardial infarction therapies include injectable hydrogels and tissue patches [26–28]. For successful cardiac patch grafting, patch bulk properties must be sufficient to integrate with the heart wall and to facilitate coordinated contraction in synchrony with the existing tissue.

### 2.2. Local Physical Properties at the Cellular Scale

In addition to bulk tissue characteristics, the porosity, fiber stiffness, and other mechanical parameters in a tissue are important to consider at the scale of the cell. One tissue engineering approach depends on the mechanical property of porosity in a significant way; replacement of damaged tissues with reseeded organ scaffolds is a promising solution to address organ supply shortages [29–32]. In the context of reseeding in tissue scaffolds, material properties such as compliance and porosity direct cellular processes at different scales. Work by several groups has helped characterized these effects, determining that nanopores (<100 nm) support the formation and crosslinking of collagen fibers, while macropores (>100 *μ*m) allow cell infiltration, distribution, and attachment [33–36]. Broadly, this is an effect of size exclusion, by which the infiltration, migration, and organization of cells within a 3D scaffold are inversely related to cell size [37, 38].

The complexity of bulk tissue mechanics must be a chief consideration in effective tissue engineering applications for the reasons described in Section 2.1. But it is important to acknowledge that stiffness of bulk tissue samples is usually much more uniform than the stiffness of individual protein fibers within them [39]. This is important because the activity of individual cells is primarily influenced by the local microenvironment even independent of the heterogeneity and organization of the bulk tissue. This may have important implications for cell and tissue function. We discuss this more in detail in Section 6 as an argument for utilization of AFM to analyze tissue characteristics.

Physiology has long considered structure function relationships linking tissue biomechanics to the cellular scale. For example, just prior to ventricular tissue contraction, end-diastolic pressure sets the initial cardiomyocyte sarcomere length [40]. The load imposed on the cell in turn determines force of contraction and modulates phosphorylation and expression of myofilament proteins such as troponin I and desmin. Because of this relationship between tissue mechanical forces and the cardiac cell, chronic changes in elasticity and stiffness may subsequently lead to maladaptive growth [41, 42].

Physical connections between cell cytoskeleton and the environment surrounding the cell facilitate force transmission intracellularly. Upon transmission, these forces affect changes in biochemical signal processes, metabolism, cell cycle activity [43, 44], circadian rhythms [45], nuclear positioning [46], invasive [47] or prometastatic [48] cancer cell behaviors, and gene expression patterns [49, 50]. These are just a few of many ways in which tissue-scale mechanics (tension, pressure, fluid shear, porosity, and anisotropy) affect cells in the local microenvironment.

Manipulation of material *ex vivo* can significantly alter both bulk and local mechanical aspects of tissue. Thus, the manipulation of material has important implications in tissue engineering applications, and it is important to characterize how biomaterials change with processing.

## 3. Decellularization in Tissue Engineering

### 3.1. Conceptualizing Decellularization and Decellularized Tissues

A tissue engineering strategy that has rapidly gained wide usage is implementation of “decellularized” tissue scaffolds or materials (e.g., hydrogels, suture, and bandages) derived from “decellularized” tissue. Decellularization is, as the name implies, the removal of cells from tissue or organs. Theoretically decellularization leaves the extracellular matrix largely intact despite disruptions to cell-matrix adhesions, thereby creating “decellularized extracellular matrix” (dECM).

After Doris Taylor's proof of concept demonstration of decellularization applied to cardiac tissue engineering, many protocols have emerged for isolating intact tissue scaffolds free of living cells. Protocol development has reflected considerations for differences in organ properties including hard versus soft tissue, organ size, and extracellular matrix composition and density. With each protocol, there is a compromise between contamination by residual intracellular contents and alterations to the extracellular matrix architecture and contents. Decellularization protocols follow a few common steps including organ isolation, chemical (or temperature) trauma to dislodge and damage living cells, and followed by several washes to remove cellular mass leaving the tissue scaffold close to the original architecture. The commercialization of decellularized tissues for therapeutic applications has led to regulation by the FDA for approved applications. A few companies in this domain include LifeCell, CryoLife, Viscus, and ACell. Decellularization protocols may involve detergent exposure, mechanical agitation of tissue, vascular perfusion, freeze-thaw cycles, osmotic shock, cell adhesion inhibition, and enzymatic digestion. In addition, these strategies have been used individually or in combination to effectively remove cell bodies from a tissue mass.

Compared to other tissue engineering materials, dECM composition contains more heterogeneity and unknown molecular components because of its high complexity, time dependence, and its variability between tissues, individuals, and species. Yet it is equally promising as it is mysterious. dECM presents distinct advantages for biological applications that make it a prime focus of the field of tissue engineering. dECM has recently become an *in vitro* model for cells in the dish and a template for tissue engineering with the advantage of retaining native biological signals and structure that are cell responsive. Initial findings using the material have been promising, and the practice of decellularization has experienced significant growth in the field of tissue engineering over the past decade (see Figure 1).

### 3.2. Recognizing Physical Changes in dECM Secondary to the Decellularization Process Itself

There is a growing appreciation that cell behavior is influenced by the physical properties of a substrate through mechanotransduction (e.g., durotaxis, whereby cells migrate spatially along a gradient of mechanical stiffness) [51–57]. The effects of cell-substrate interactions depend on both the cell type and the ECM components. This is because different cell types express unique subsets of integrins, integrins bind differentially to matrix proteins based on their subtype, and each integrin subtype induces different signaling cascades within the cell [14, 58]. The importance of material physical properties for biological applications has never been more apparent.

Nonetheless, many cell substrates used in tissue engineering research (dECM included,) present different mechanical stimuli to cells than what is present in the native ECM microenvironment. For example, the commonly used cell culture substrate acrylamide shows a mostly linear mechanical response with increasing strain, which is unlike typical ECM strain-stiffening behavior [14].

Changes in fiber thickness, which may result from the method of decellularization, could have important effects on tensile, elastic, and bending properties of dECM materials. In theory, characteristic differences in ECM mechanics secondary to decellularization processing can produce confusing results. Empirical observations on decellularized matrix may deviate from typical cell behaviors in situ, obscuring the relevance of any scientific findings.

In practice, we do note some examples whereby it has been demonstrated that tissue decellularization can significantly affect at least bulk tissue compliance. Decellularization has resulted in reduced lung elastic modulus, attributed in part to loss of elastin content in the tissue [59]. Mechanical strength of fibroblast cell sheets significantly decreases following treatment with SDS for cell removal [60]. Porcine aortic valves exhibit greater extensibility and a loss of stiffness after decellularization by a variety of methods [61]. Despite these few examples which demonstrate a weakening of mechanical integrity following decellularization, dECM remains a particularly attractive material for tissue engineering. This is because in addition to its ability to present key biochemical elements, it also may retain structural and mechanical features that are important to cells. To some degree, structural and mechanical preservation is impacted by the particular decellularization technique used. The extent to which retention of material properties through decellularization happens is generally not well described in studies that employ dECM. Use of analytic techniques such as AFM, FTIR, and DMA can be done pre- and postdecellularization to determine the extent of any deleterious effects of tissue processing.

### 3.3. Changes in Cardiac dECM Warranting Further Attention

To provide further granularity to our last discussion point (Section 3.2), we will discuss further using the example of cardiac extracellular matrix, a familiar material in our lab.

We recently investigated cooperative signaling of cardiac extracellular matrix and mechanical signals independent of structure [62]. Solubilizing dECM into solution allows receptor-ligand interactions to be examined with restricted spatial cues. Similarly, using synthetic elastic substrates *in vitro* and exogenous plant-derived crosslinking agents replicates mechanical cues with limited direct biological signaling in mammalian systems.

The heart is a challenging target for regenerative therapies because of the postnatal terminal differentiation of cardiomyocytes. Recognizing the robust proliferative nature of fetal development, we and others have shown that introducing fetal dECM to primary cardiomyocytes prolongs the mitotic signaling of postnatal cardiomyocytes *in vitro*. The dECM can be delivered solubilized into media or as a monolayer to plate cells on. Thermal polymerization allows dECM to be delivered as a liquid that forms a hydrogel at body temperature. Comparative delivery of age-sourced dECM into injured mice demonstrates a proregenerative phenotype after surgical infarction with fetal ECM and low to antiregenerative phenotypes with adult-sourced dECM. Homology of dECM proteins has allowed these experiments to be conducted with both allogenic and xenogeneic transplants with similar results [7, 63]. Proteomic analysis of the decellularized hearts by the Lauren Black lab and others suggest that there are compositional differences in the cardiac dECM with aging and more distinctly with disease [64]. Agrin is one of the proteins enriched in the dECM that has been shown to regulate mitosis-related yes-associated protein 1 (YAP) activity in cardiomyocytes. There are several groups focused on optimizing proteomic profiling of the dECM in the heart and other tissues [65–67]. The degree of cellular contamination and extracellular loss of the decellularized tissue continues to be an important focus as well.

**Figure 2:**
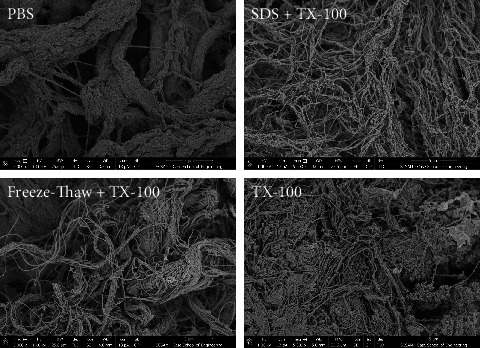
The structures of decellularized liver ECM (top right and bottom quadrants) are noticeably different by various protocols (e.g., in porosity, fiber thickness, and bundle fiber thickness). Native and decellularized porcine liver samples were prepared for imaging by drying with serial ethanol immersion, followed by critical point dryer, and then sputter coating with 3 nm thickness palladium using a Denton Desk IV vacuum system. SEM images were taken at 5,000x magnification using Helios NanoLab 650.

Our work suggests the decellularization approach to isolating the dECM is critical [62]. We have also noted that there is higher biological potency of the decellularized tissue when digested to an intermediate stage rather than complete solubilization into a *bonafide* solution. While reduced potency could be due in part to digestion of putative signaling regions of peptides, the colloidal suspension of the partial digested tissue suggests that intact microparticles of decellularized tissue retain some tertiary structure that enhances their cell signaling effects. Western blotting suggests that protein integrity of small proteins such as agrin is not significantly reduced by full digestion giving some support to protein aggregates and tertiary structure playing a role in the higher potency of the partially digested dECM. From a material science perspective, particle analysis, electron microscopy, and cryo-EM could yield critical information to the structure function of dECM microparticles (to be discussed further). Distinguishing the potency of suspended particles relative to proteins in solution strengthens the importance to the structure of aggregate microdomains relative to individual proteins and peptides. As shown in Figure 2, the decellularization method significantly affects structural features (fiber thickness, porosity, and fiber networking). It remains to be determined if gelation and biological signaling is also impacted by these changes.

Mechanical signaling is an important molecular player in dECM-mediated signaling. We observe that decreasing the microenvironment stiffness of the heart improves the fetal dECM-induced heart regeneration in neonatal mice. We developed a method to tune mouse heart stiffness by intraperitoneal injection of crosslinking regulators. Genipin, a plant-derived protein crosslinker, increased the elastic modulus of the decellularized heart by 3-fold, and *β*-aminopropionitrile (BAPN), a lysyl oxidase inhibitor derived from pea plants, reduced the elastic modulus to an order lower. Similar results are achieved in the dish with BAPN and ribose (or genipin) with tissue explants.

### 3.4. Physical ECM Changes in the Setting of Disease

Mechanical features of the extracellular matrix are gaining more recognition in disease progression. This recognition provides further motivation for the development and application of mechanical profiling techniques to ECM and decellularized tissue for the study of disease pathogenesis. A novel inflammatory role for mechanical signals was recently investigated in the retina by the Yang group. It was found that in early stages, increased basement membrane stiffness may exacerbate or even replace circulating inflammatory signals as a cause of worsening diabetic retinopathy [68]. Traditionally, the pathogenesis of diabetic retinopathy has been recognized to involve a complex interplay of biochemical mechanisms induced by hyperglycemia with subsequent inflammation and vascular permeability changes [69]. The inflammatory and vascular changes are facilitated by activation of retinal endothelial cells in response to hyperglycemia; activated cells increase ICAM-1 expression which promotes leukocyte-endothelial cell adhesion, increasing local inflammation. Hyperglycemia also increases lysyl oxidase expression which increases the basement membrane stiffness by integrating new collagen into the ECM [70]. Yang et al. found that upon inhibition of lysyl oxidase, basement membrane stiffness is reduced, in turn suppressing endothelial cell activation [68]. Traditionally, studies and therapies have focused predominantly on the role of soluble factor-induced endothelial cell activation without considering the role of membrane stiffness. Work done by Yang et al. shows that membrane stiffness, independent of hyperglycemia, is necessary and sufficient to promote endothelial cell activation, a key step in the progression of diabetic retinopathy. This provides a rationale for investigating the extracellular environment as a therapeutic target for diabetes-induced retinal disease. Moreover, it may be inferred from these findings that basement membrane mechanics can play a significant role in disease progression in other tissues. Further investigation of mechanically induced disease progression in other organs may be warranted.

For tissue engineering, characterization of the mechanical state of decellularized tissues and basement membrane may be critical to the response of donor cells on tissue scaffolds derived from animals or patients.

## 4. Assessing the Impact of Decellularization Technique on End-Product

### 4.1. Porosity

The particular decellularization methods used can influence dECM physical properties. For example, consider porosity: it has been shown that the use of ionic, rather than an anionic detergent, may result in significantly more porous dECM [71]. It has also been shown that inclusion of the ion chelator EDTA may produce a less porous dECM [72].

In order to quantify the porosity of scaffolds derived from various decellularization approaches, several tools exist, including mercury porosimetry and electron microscopy. Characterization of scaffold pore size is a useful tool for determining changes from the native biological system. Therefore, for studies concerned with maintaining the reseeding potential of a decellularized tissue, it will be important to characterize various decellularization strategies on the basis of the change in pore size which they induce. Still, even as scaffold porosity is understood to direct cellular organization after seeding, only few studies have noted concern regarding the porosity changes observed in decellularized tissues used as cell scaffolds.

### 4.2. Diffusivity

Similarly, various tissue decellularization strategies have been demonstrated to result in changes in tissue diffusivity [73]. This is most clearly represented by differential swelling of tissues decellularized by different protocols. Because cells rely on the diffusion of soluble factors and nutrients into their local microenvironment, the diffusive capacity of a material is integral to the construction of usable cell scaffolds. When elements of the cellular microenvironment, such as the diffusivity of growth factors and nutritive molecules, are altered, adverse effects on cell behavior are observed [73]. This phenomenon is well described during the progression of tumors which rely on increased diffusion to meet the oxygen and nutrition requirements of rapidly dividing cancer cells. Consequently, decellularization processes which increase tissue diffusivity may provide an apt model for the study of diffuse disease states, but do not appropriately retain features of the healthy microenvironment. Therefore, moving forward, it will be worthwhile to investigate the relative diffusivity of tissues decellularized by different protocols, either by FTIR or histological analysis.

### 4.3. Spatial Structure

Among the changes in porosity and diffusivity which may occur as a result of decellularization, changes in the spatial environment within which cells adhere, grow, and signal occur as well. As a result, it is possible that the anchorage-dependent signaling (and consequently the phenotype) of cells will be altered. Such an effect is observable in cases of traumatic events such as hypertrophy wherein cell swelling alters cellular attachment and organization, resulting in positive force on the cell and rendering a hypertrophic phenotype [74]. Moreover, cell culture constrained to areas of varying size has previously demonstrated impact on cell organization and morphology, indicating that spatial manipulation in cell culture regulates cell growth and viability [75]. As popular decellularization methods rely on cell swelling and rupture, the architectural framework of the extracellular environment may be changed. Specifically, swelling cells may impart positive force on their environment, thereby enlarging the spaces into which new cells will be seeded. Consequently, the potential for reseeding and maintenance of normal cell behavior within a decellularized scaffold may be altered. In order to determine the magnitude of these alterations, it will be useful to analyze samples of nondecellularized tissue, of decellularized tissue, and of reseeded tissue. This can be achieved by electron microscopy to compare the quantity and orientation of cells in each group. Additionally, proteomic profiling of each group may provide insight into the expressions of cells which are lost or retained upon introduction to a decellularized tissue. Ultimately, the utilization of decellularization techniques which minimize changes to the spatial microenvironment within a scaffold may improve anchorage-dependent signaling and phenotype of reseeded cells.

Recently, we have even found some evidence that decellularization method affects structural characteristics of the matrix in our own work. Incorporating repeated freeze-thaw thaw cycles into a detergent-based liver decellularization protocol resulted in dECM with significantly different protein fiber sizes than native liver tissue on average, while strictly detergent-based strategies did not have this effect (unpublished data). All decellularization techniques investigated produced ECM of qualitatively altered structure compared to native ECM (see Figure 2). We observe distinct observable changes in porosity, individual fiber thickness, bundled fiber thickness, nonfibrous structures, and effective fiber length. The impact of these changes is yet to be determined; however, we anticipate independent of altered protein composition that mechanical and topographical differences would have some effect on adhesion and biological activity of reseeded cells.

## 5. Characterizing Matrix Changes When Protocol Variability Is High

Despite the fact that decellularization method can substantially change material properties, it is difficult to draw conclusions about the relationship between many of the mechanical parameters and the decellularization techniques/protocols that were used. Some notable exceptions exist, e.g., the use of glutaraldehyde or UV light to facilitate matrix crosslinking and increase stiffness. Examples such as this are few and far between. Physical properties in published decellularization papers are often lacking. Analyses of biochemical composition are more common. Further implementation of underused analytic techniques to better characterize dECM would be informative.

Of note, it may be possible to infer physical property changes from observations of biochemical change in the matrix. For example, the attachment of glycans to proteins is critical for many physiological phenomena, including ECM crosslinking. Matrix crosslinking is associated with stiffer ECM, so the removal of these sugar moieties could impact both matrix-matrix and cell-matrix interactions by contributing to matrix softening. Whether or not glycosylation levels are actually affected by decellularization usually goes unchecked. While assessing the effects of decellularization on glycosylation may be meaningful for other reasons, there are more direct methods to measure the effects of decellularization on ECM stiffness.

Extensive research has been done to strengthen our understanding of organizational and physical effects on cells. The strong relationship between matrix mechanics and cell activity motivates a thorough assessment of matrix properties. Two chief scientific concerns, experimental outcomes *in vitro* and performance *in vivo*, are impacted by the pairing of matrix and cells via mechanical cues. Underutilized techniques, described in the next section, can help to assess the quality and extent of this impact. The described analytic techniques would be particularly useful since decellularized matrix material is protocol-dependent and thus may be significantly variable from one lab to the next.

## 6. Adding New Techniques to the Current “Standard of Practice” for Matrix Characterization

While analysis of dECM physical properties is uncommon, it is sometimes done. In the remainder of this review, we highlight some successful analytical techniques that have been used to characterize dECM physical properties, with the hope that this inspires further analysis of physical properties in future dECM studies. These techniques are summarized and organized in Table 1.

### 6.1. Atomic Force Microscopy (AFM)

Multiple methods exist to detect differences in stiffness of dECM. One such technique that has been successful is AFM. In one prior study, after chemical damage was induced in mouse livers, AFM was used to show a significant and measurable increase in stiffness in the liver postdecellularization [76]. AFM has also been used successfully to measure stiffness of the decellularized pig hearts [77] and decellularized human livers [4]. While AFM may be usefully applied to soft tissues, uniaxial tensile and/or compressive testing can also be effective for other decellularized samples. This has been demonstrated with electrospun porcine lung matrix scaffolds [78], decellularized connective tissue [79], and with decellularized kidney [80] and liver [81].

The ability to compare material stiffnesses from different regions within an organ is one of the major advantages of AFM. AFM has been applied with success to decellularized tissues in several cases, revealing that local stiffness depends significantly on not only region within tissue but also on decellularization process. The effect of decellularization process on mechanics also varies between tissue structures within the organ. For example, by AFM assessment of decellularized alveolar septa, alveolar junctions, pleura, vessels' tunica intima, and tunica adventitia, it was found that stiffness was reduced by on average 35% with CHAPS detergent, compared to SDS, and out of all the sites assessed, this difference was most pronounced in the vessels' tunica intima [82]. Another lung dECM study utilizing AFM found pleural regions were threefold stiffer than alveolar walls [83]. Regional AFM analysis was similarly done for decellularized, native, and frozen aortic vessels, revealing enhanced strength in some layers of the vessels (tunica media and adventitia), but reduced mechanical properties in the lumen after decellularization [84].

In many tissue engineering applications, local differences in tissue stiffness would have meaningful impact on migration and regulation of seeded cells. Thus, one could argue that different decellularization methods (or post hoc strategies to correct any mechanical perturbations introduced) may be needed for different parts of a heterogeneous organ scaffold. To this end, AFM analyses would have great utility. The abovementioned studies demonstrate the feasibility of using AFM to identify regions of decellularized tissue where mechanical strengthening (e.g., by crosslinking agents) may be desired.

Several other interesting findings have come to light through the application of AFM to decellularized tissues. AFM has been used to quantify distinct stiffness changes over time in the regenerating zebrafish heart extracellular matrix, enabling comparison of stiffness for noninjured myocardium with the regenerating wound area in decellularized samples [85]. By using AFM, Garcia-Puig et al. found no significant differences between wound and noninjured area at 7, 14, or 30 days after injury. Future AFM studies comparing this finding to myocardial injury models from other species may point to ECM stiffness as an important factor that contributes to the great capacity for myocardial regeneration in zebrafish.

In another recent dECM study, AFM was used to measure differences in stiffness at various steps in the tissue engineering process, including precell seeding, with cells seeded, and postcell removal, in liquid or dried environments [86]. This same study highlights another significant advantage of AFM: in conjunction with mechanical stiffness properties, researchers using AFM can assess topographical features within the same material sample. AFM mechanical and topographical mapping of decellularized scaffolds by Viji Babu et al. revealed that three distinct types of fibroblasts (normal, scar, and Dupuytren-derived) each exhibited regular shape, spreading, and cytoskeletal organization when cultured on each of five matrices. Yet Young's moduli of normal fibroblast populated scaffolds were lower than those of scar- and Dupuytren's fibroblast-populated scaffolds. Histograms generated from the stiffness mapping of the samples illustrated mechanical heterogeneity across the samples. On acellular dermis samples, thicker and uneven fibers (0.7-0.9 *μ*m thickness) were observed postculture with scar or Dupuytren's fibroblasts, but not on samples that had had normal fibroblasts [86].

As mentioned, we have recently had success applying AFM to demonstrate significant stiffness changes between control, BAPN-, and ribose-treated decellularized cardiac explants [62]. Specifically, we were able to assess the stiffness of cardiac dECM in an in vivo-like hydrated environment by first adhering the tissue to petri dishes, and then immersing samples in PBS for the measurement. It should be noted again that the altered organs or explants were decellularized before this AFM analysis. BAPN was shown to inhibit matrix remodeling, effectively resulting in a half-as-stiff tissue, while ribose enhanced collagen and elastin matrix integration, thereby stiffening the cardiac ECM more than 3-fold. Delivery of exogenous fetal cardiac dECM was found to increase cardiac function and result in decreased fibrosis 3 weeks after myocardial infarction (MI).

Though not the focus of this study, we were also able to assess elastic modulus and topographical height heterogeneity across cardiac explant sample surfaces via AFM technology. We found that control samples exhibited a range of local stiffnesses (64 ± 37 kPa, *n* = 2977), while the BAPN- and ribose-treated samples ranged in stiffness from 27 ± 35 kPa (*n* = 2996) and 197 ± 167 kPa (*n* = 4537), respectively [62]. Individual cardiomyocyte activity is modulated by paracrine activity from endothelial cells [87] and also likely local stiffness parameters [88–91]. Therefore, theoretically, two tissues with the same mean elastic modulus may behave quite differently if one were to have a narrow Gaussian distribution about the mean, while the other has a widened or even bimodal distribution of elastic moduli. Thus, the pattern of distribution of stiffnesses across the sample, as can be demonstrated by AFM, may impact cardiac scar formation and contractile performance post-MI and is potentially a focus of future study. Tissue decellularized by perfusion strategies, in particular, may exhibit unusual elastic parameter distribution since well-vascularized regions of the material are subjected to higher flow rates, pressures, and detergent concentrations compared to poorly vascularized spaces.

AFM-derived mechanical tissue parameters may not resemble other values described for the heart for normal physiology; however, the mechanical analysis of the heart and other tissues warrants renewed evaluation. AFM analysis of actively contracting the heart compared to cardiac tissue arrested in a relaxed or contracted state may illuminate key characteristic differences; additionally, well-functioning cardiac regions can be probed and compared against hypokinetic or even akinetic regions. Decellularization method and orientation of the AFM probe to the sample features are both factors that may affect AFM results. As suggested by the morphology of tissues in Figure 2, one might imagine changes in ECM structure may obscure results due potentially to fiber entanglements, stiction, and porosity altering AFM probe release.

AFM is a powerful technique with important application in the analysis of decellularized matrix regional stiffness and topography. The increased data granularity compared to DMA, rheology, and tensile or compressive testing is a unique advantage of AFM. Some recent dECM studies have already demonstrated this, and improvements to the technology will likely make its application to tissue engineering even more straightforward in the coming years. Recent adaptations have already been made to the method that improves sensitivity over a wider length scale relevant to that of cells-tissues [92].

### 6.2. Dynamic Mechanical Analysis (DMA)

DMA proffers researchers with a nondestructive material test. It is frequently used to assess stress response of polymers with varying frequency loads, but it is also useful for biological samples. In addition to stiffness, creep, and relaxation tests, DMA can also be used to determine storage and loss moduli—descriptors of material response to dynamic oscillating loads recently hypothesized to be an important feature of matrix-cell communication [93]. Young's modulus, storage, and loss moduli have been determined for both human and porcine liver using DMA [94]. Various hydrogels have been assessed by DMA. DMA has allowed researchers to quantify the effect on mechanics of adjunct particles incorporated into gels [95] and to compare the dynamic behaviors of gels with varying gelatin to elastin ratios and with varying crosslinking degrees [96]. It is a unique tool for the field of tissue engineering that has seldom been utilized for decellularized matrix analysis.

Sparse use of DMA for decellularized tissues may in part be due to reproducibility challenges related to the soft character of biological specimens. Mechanical characterization by DMA, however, has been shown to be highly reproducible if tissue sample dimensions are first optimized. While more accurate loading profiles are generally obtained when thickness/surface area ratio is high, sample slippage can occur if surface area is inadequate. Ramadan et al. found reproducible storage and loss moduli with use of 8 × 8 × 4 mm myocardial slabs loaded into a DMA apparatus that had a 10 × 10 × 5 mm space limitation [97]. By using DMA, the authors were able to demonstrate linear stiffness, storage, and loss moduli increases as loading frequency increases within the physiologic frequency range of the heart (0.5 to 3.5 Hz, corresponding to 30 to 210 beats per minute) [97]. DMA provides highly relevant data for dynamic organs subjected to oscillating loads.

Many, if not all, organs targeted by tissue engineering efforts are subjected to dynamic loading over a range of frequencies. But perhaps the most prominent example of this is the beating heart. Clinical data has suggested that after acute myocardial infarction, elevated discharge heart rates are associated with increased mortality risk within 1-3 years [98, 99]. The causality in this association is unclear and likely complicated, but presumably, increased cardiac tissue stiffness and decreased contractile functions associated with increased heart rate contribute to the increased mortality rates observed.

Alteration to dECM dynamic mechanical performance as its composition and organization become remodeled by cells seeded within it is an important tissue engineering consideration. Cell-induced alterations are reflected by temporal changes in complex modulus and phase angle over a frequency sweep with DMA. For example, Mercuri et al. demonstrated increase in decellularized nucleus pulposus hydrogel complex modulus with increase in frequency over the range of 0.1 to 40 Hz [100]. Fourteen days after seeding with stem cells, complex modulus was higher at all frequencies if the scaffold had been immersed in nucleus pulposus differentiation media. By contrast, complex modulus was only higher compared to nonseeded controls at high frequencies (10 and 40 Hz) if the scaffolds were kept in standard media [100]. DMA enables a better appreciation of cell contributions over a range of loading frequencies to which biologic tissue is subjected.

Few other examples of DMA in decellularized matrix studies exist, but tangential studies on hydrogels and minimally processed organ tissues demonstrate that it could be a useful technique applied to dECM in the future—especially due to the dynamic oscillatory nature of the tissues in which dECM is applied.

### 6.3. Electron Microscopy (including Cryo-EM, SEM, and TEM)

Another useful tool for characterizing the physical composition of dECM is cryogenic electron microscopy (cryo-EM), which provides data describing the spatial organization of molecules within a sample. This is achieved by cryogenically freezing and thinly sectioning a sample, dECM in this case. Next, standard scanning electron microscopy (SEM) or transmission electron microscopy (TEM) approaches may be employed. In SEM, a beam of electrons is directed at the sample, and data regarding the angle at which the beam is reflected along various points of the sample is aggregated to form a three-dimensional image describing the surface topography of the sample. In TEM, a beam of electrons is transmitted through the sample to form a negative two-dimensional rendering of the sample, outlining the size, shape, and spatial organization of molecules in the sample. From this image, a three-dimensional representation of the sample can be extrapolated. By comparison to nuclear magnetic resonance (NMR) spectrometry or X-ray crystallography (XRC), this approach is better suited for large, heterogeneously complex samples.

Electron microscopy (EM) has advanced to become a principle technique of structural biology for nanometer spatial resolution images of membrane proteins, macromolecular complexes, and a wide range of biological samples. EM analysis occurs in high vacuum conditions requiring fixed and dry sample preparation leading to shrinkage and morphological alterations. Cryo-electron microscopy circumvents topographical artifacts by preserving ultrastructure integrity using cryogenic approaches. We found only one published cryo-EM application to image decellularization-produced ECM scaffolds. Fernández-Pérez and Ahearne showed qualitative retention of fibrillary and porous structures by cryo-EM after decellularization of porcine corneas [101]. Decellularization can impact structural properties, and cryo-EM offers a high resolution for comparative analysis of fresh biological samples and decellularized tissues.

In our review of decellularization papers, we found that scanning electron microscopy (SEM) was the most frequently employed technique for dECM structural analysis. Once decellularized matrix samples are prepared and imaged, SEM images can be analyzed to gain information about a variety of material characteristics, including fiber organization/alignment, feature size, porosity, node density, and particle morphology. See Table 1. In practice, assumptions about sample uniformity may isolate smaller imaging regions of a sample as representative of the whole decellularized tissue. We could not find any articles that profile ultrastructure features throughout a sample. Generalization of material properties from analysis of a small region of the tissue may lead to erroneous conclusions about characteristics such as material porosity or feature size and organization. Increasing the number of regions imaged per sample can help to curb, but may not eliminate, errors of this type.

### 6.4. Additional Techniques

Fortunately, complementary techniques to SEM exist for quantification of material porosity, density, and particle size. CT scanning has been an effective method for nondestructively quantifying density of decellularizing rat liver, which can be used as a marker for cell removal [102]. CT scans have also been used to gain insight into the architecture, porosity, and the surface: volume ratio of decellularized bone matrix [103]. X-ray imaging is another tool which can provide information about material structure and density [72, 103].

As another alternative to SEM, dynamic light scattering techniques can be used to measure particle size. This has been demonstrated for both decellularized zebrafish and mouse heart matrix [7], as well as for decellularized porcine lung matrix [104].

Measuring the displacement of ethanol by extended immersion of a cell-free scaffold in it can be an effective method for quantification of material porosity [78]. Mercury porosimetry, which would involve intrusion of mercury at high pressure into dECM, could also theoretically be applied to quantify dECM porosity. This technique is most amenable to hard tissues (particularly bone), since the high pressure may damage softer tissues [105].

Mass spectrometry can be used to measure the stoichiometry and composition of protein complexes. There are some references of mass spectrometry applied to decellularized biological samples for molecular imaging as well as molecular compositional analysis. We found at least one publication where localization of ECM proteins was profiled with MALDI mass spec to confirm the integrity retention of specific decellularization protocols. The more prevalent application of mass spectrometry is for molecular composition (independent of structure and spatial localization).

Stiffness characterization can also be done by rheological methods. Increases in shear storage, loss modulus, and stiffness have all been detected in carbon tetrachloride-induced fibrotic rat livers using rheological methods on the tissue after it had been decellularized [81]. By rheology, storage and loss moduli of dECM hydrogels produced by varying protocols can also be effectively compared [101]. In our lab, rheometry has been useful in quantifying polyacrylamide substrate stiffnesses of varying acrylamide to bis-acrylamide ratios. Rheometry has enabled us to demonstrate the effect modification of fetal and adult cardiac matrix biomolecules on cardiomyocyte cell cycle activity plated on varying stiffness substrates in a recent study [62]. No significant differences were found in BrdU-positive cardiomyocytes on different polyacrylamide stiffnesses in the absence of dECM. However, when cardiac ECM was added to the culture, differences were found in BrdU activity between various rheologically quantified stiffness substrates [62]. Rheology, including rheometry and viscometry, is useful for more than just quantification of elastic, storage, and loss moduli. It can also be used to track gelation kinetics of decellularized matrix materials with changes in temperature [5]. Few studies have examined the elastic properties of decellularized tissues. The relaxation of cardiomyocyte relative to the surrounding basement membrane, or converse resistance for a contracting myocyte (or fibroblast), would alter mechanosensitive machinery including stretch activated channels [106]. Application of rheometers beyond nanoindentation to examine shearing and elasticity provides biophysical information relevant to cell interaction with basement membrane.

Of note, one creative alternative method for gelation kinetics analysis is turbidimetric spectrophotometric analysis, whereby gels are cast into a plate at 4°C, then warmed to 37°C on a plate reader, while absorbance values are measured every few minutes [101, 107]. Based on absorbance measurements, the progression of gelation over time can then be calculated and tracked.

While there exist many examples that physical and structural material characterization techniques can be applied to dECM materials (Table 1), their inclusion is not standard in studies that use dECM. Incomplete characterization of the materials used in decellularized matrix studies can introduce frustrating challenges for researchers who hope to compare incongruent results from different, but similar studies.

Venkatraman group at Nanyang Technological University presented the only paper we could find with multiple bulk biophysical tests to quantitatively assess functionality of decellularized scaffolds [108]. The authors hypothesized that mesenchymal cell reseeding would replenish structural and compositional features of ECM scaffolds depleted in the decellularization process. Surface properties were measured by FTIR and SEM to demonstrate molecular cloaking of detergent-induced GAGs exposure and scaffold porosity. Tensile mechanical testing was applied to confirm bulk stiffness recovery. In addition, the investigators applied thermogalvanometric and calorimetry to examine water content and thermal profiling.

## 7. Conclusion

Physical properties of dECM can have important implications when placed in a biological context. While the literature primarily focuses on the biochemical properties of dECM, this review takes particular interest in the lesser-explored mechanical characterization of dECM. The mechanical cues which cells derive from their native ECM environments will be critical to functionality of *in vitro* experimental models and dECM-based tissue engineering.

The extracellular matrix (ECM) is a complex mesh of structural proteins, proteoglycans, and adhesive proteins which maintains the structural support of biological tissues and provides cues directing the behavior of cells within them. For tissue engineering, many substitute cell mesh materials exist, each exhibiting a unique range of physical, structural, and chemical cues.

Studies that use dECM should therefore perform some physical characterization of the tissue-derived material. Analysis of dECM preparations' physical properties may become increasingly valued as the appreciation for how mechanical forces and substrate mechanics influence cell behavior continues to grow. In future studies where dECM is applied to cancer and other pathologic states, its material properties should be analyzed and tuned to ensure that they accurately reflect the altered microenvironment, such as in a tumor, that the application typically exhibits.

For years, techniques such as mass spectrometry, western blots, histology, and immunohistochemistry have proven to be useful in characterizing dECM. Many powerful, but less-commonly-used analytic, techniques also exist, such as rheometry, AFM, DMA, SEM, and FTIR. The working range of these instrumentation and operating procedures may require modifications to facilitate analysis of biological tissues which can be softer and smaller than common samples studied. Furthermore, the natural wet state of biological tissues may need to be maintained for accurate analysis in some cases. By more frequent inclusion of some of these techniques in future decellularization studies, the qualities of dECM, and how these qualities impact cell behavior, may be better understood. We predict that the growing knowledge of substrate mechanics' influence on cell behavior will drive such analyses.

## Figures and Tables

**Table 1:** Physical material property characterization techniques for dECM.

Method of analysis	Material properties of interest	Description of sample analyzed
Atomic force microscopy (AFM)	Young's modulus	Native and decellularized human liver cubes [4]; decellularized and native pig myocardium layers [77]⁠; decellularized rat lung tissue [83]; decellularized mouse heart tissue [62]
Rheology	Shear storage, loss moduli, Young's modulus	Normal and fibrotic decellularized rat livers [81]⁠, native and decellularized pig cornea hydrogels [101]
	Steady sheer viscosity, storage modulus, gelation kinetics	Liver ECM hydrogels (various species) [5]
SEM	Fiber organization/scaffold structure	Native and decellularized human liver cubes [4, 109]⁠; native and decellularized zebrafish and mouse heart matrix [7]⁠; native and decellularized pig myocardium layers [77]⁠; native and decellularized rat kidney [80, 110]⁠; pig myocardial matrix gel [111]⁠; liver ECM hydrogels (various species) [5]; decellularized human umbilical cord Wharton's Jelly [112]; native and decellularized rat liver [72]⁠; native and decellularized rat skeletal muscle [113]⁠; native and decellularized mouse lungs [114]; native and decellularized pig lung matrix [104]
	Fiber diameter/particle sizing	Liver ECM hydrogels (various species) [5]⁠; native and decellularized pig lung matrix [104]; cryoground decellularized pig cartilage matrix [115]⁠; electrospun decellularized pig lung matrix scaffolds [78]⁠
	Porosity	Liver ECM hydrogels (various species) [5]⁠; native and decellularized rat liver [72]⁠
Liquid displacement	Porosity	Electrospun decellularized pig lung matrix scaffolds [78]⁠
Uniaxial tensile testing	Young's modulus	Decellularized and native kidney tissue [80]; electrospun decellularized pig lung matrix scaffolds [78]⁠; decellularized and native pig enthesis connective tissue [79]⁠
	Maximum force/tensile strength, maximum elongation	Decellularized and native pig enthesis connective tissue [79]⁠; decellularized and native kidney tissue [80]
Uniaxial compressive test	Elasticity	Normal and fibrotic decellularized rat livers [81]⁠
CT scan	Density	Decellularizing rat liver tissue [102]⁠
	Architecture, surface density, porosity, surface : volume ratio	Native and decellularized human bone cubes [103]⁠
Sessile drop technique	Surface wettability	Electrospun decellularized pig lung matrix scaffolds [78]⁠
X-ray imaging	Structure, density	Native and decellularized rat liver [72]⁠
	Mineral phase crystallinity, bone mineral density	Native and decellularized human bone cubes [103]⁠
Dynamic mechanical analysis (DMA)	Storage and loss moduli, Young's modulus	Human and pig liver [94]⁠
Extended buffer immersion	Degradation	Electrospun decellularized pig lung matrix scaffolds [78]⁠, decellularized pig heart matrix gel [62]
Image quantification	Structural collapse	Native and decellularized human and pig livers [116]⁠
Bulk tissue mechanics	Static and dynamic elastance	Native and decellularized mouse lungs [114]⁠
Dynamic light scattering (DLS)	Particle size/radius	Native and decellularized zebrafish and mouse heart matrix [7]⁠; native and decellularized pig lung matrix [104]⁠
Cryo-EM	Fiber organization/scaffold structure	Native and decellularized pig cornea hydrogels [101]
Viscometry	Viscosity	
Mercury porosimetry	Porosity	
Optical tweezers	Microviscosity surrounding a particle	
Differential scanning calorimetry (DSC)	Thermal properties—glass transition temp/crystalline melting temp	
Contact profilometry	Surface feature profile, material roughness	
